# Threshold for Pre-existing Antibody Levels Limiting Transduction Efficiency of Systemic rAAV9 Gene Delivery: Relevance for Translation

**DOI:** 10.1016/j.omtm.2019.04.004

**Published:** 2019-04-19

**Authors:** Aaron S. Meadows, Ricardo J. Pineda, Laurie Goodchild, Tierra A. Bobo, Haiyan Fu

**Affiliations:** 1 Center for Gene Therapy, Research Institute at Nationwide Children’s Hospital, Columbus, OH, USA; 2 Vivarium, Research Institute at Nationwide Children’s Hospital, Columbus, OH, USA; 3 Department of Pediatrics, Division of Genetics and Metabolism, Gene Therapy Center, School of Medicine, University of North Carolina at Chapel Hill, Chapel Hill, NC, USA

## Abstract

Widespread anti-AAV antibodies (Abs) in humans pose a critical challenge for the translation of AAV gene therapies, limiting patient eligibility. In this study, non-human primates (NHPs) with pre-existing αAAV Abs were used to investigate the impact of αAAV9 Ab levels on the transduction efficiency of rAAV9 via systemic delivery. No significant differences were observed in vector genome (vg) biodistribution in animals with ≤1:400 total serum αAAV9-IgG compared to αAAV9-Ab-negative animals, following an intravenous (i.v.) rAAV9-h*NAGLU*^op^ (codon-optimized human α-N-acetylglucosaminidase coding sequence cDNA) injection. Serum αAAV9-IgG at >1:400 resulted in a >200-fold decrease in vg in the liver, but had no significant effect on vg levels in brain and most of the peripheral tissues. Although tissue NAGLU activities declined significantly, they remained above endogenous levels. Notably, there were higher vg copies but lower NAGLU activity in the spleen in NHPs with >1:400 αAAV9 Abs than in those with ≤1:400 Abs. We demonstrate here the presence of a threshold of pre-existing αAAV9 Abs for diminishing the transduction of i.v.-delivered AAV vectors, supporting the expansion of patient eligibility for systemic rAAV treatments. Our data also indicate that high pre-existing αAAV9 Abs may promote phagocytosis and that phagocytized vectors are not processed for transgene expression, suggesting that effectively suppressing innate immunity may have positive impacts on transduction efficiency in individuals with high Ab titers.

## Introduction

Adeno-associated virus (AAV) vectors are promising as effective gene-delivery tools for long-term transduction in a broad range of tissues. They have displayed efficacy and safety after systemic delivery in numerous pre-clinical disease models and in clinical trials, particularly for monogenic diseases.^1^, ^2^, ^3^, ^4^, ^5^, ^6^ Recognition of the trans-blood-brain barrier neurotropic properties of the AAV9 vector^7^, ^8^ has led to significant advancements in AAV gene delivery for diseases with global or broad neuropathy in the CNS, demonstrating promising clinical potential.^4^, ^5^, ^6^ These studies have led to the translation of systemic AAV9 gene delivery to a phase I clinical trial in patients with type 1 spinal muscular atrophy (SMA1; ClinicalTrials.gov: NCT02122952);^9^ phase 1/2 trials in patients with MPS IIIA (ClinicalTrials.gov: NCT02716246),^10^ MPS IIIB (ClinicalTrials.gov: NCT03315182),^5^, ^11^, ^12^ and MPS II (IND17838);^13^ and intrathecal gene-delivery clinical trials in patients with giant axonal neuropathy (NCT02362438)^14^ and Batten disease (CLN6; ClinicalTrials.gov: NCT02725580).

As effective AAV gene therapy approaches become available for clinical application, pre-existing host humoral immunity against AAV poses critical challenges. Although it has no known pathogenesis, AAV is widespread in humans, and more than 90% of the adult population is naturally infected, with a high prevalence of antibodies (Abs) to various AAV serotypes.^15^, ^16^ Although AAV2 is the most prevalent serotype in humans, cross-reactivity among different serotypes^15^, ^16^, ^17^, ^18^ reduces the potential utility of AAV vectors packaged in capsids of alternative serotypes. Anti-AAV Abs (αAAV Abs) also arise following recombinant AAV (rAAV) gene delivery, making re-administration unfeasible. Various clinically relevant models have been established to characterize anti-AAV humoral immunity and cross-reactivity. 17, 19, 20, 21, 22, 23 No effective approaches are currently available to overcome pre-existing αAAV Abs, which diminish the efficacy of systemically delivered rAAV vectors and broadly limit their application in terms of patient eligibility in clinical trials. The prevalence of pre-existing αAAV Abs is high in humans, but the serum levels vary greatly among individuals.^15^, ^16^, ^24^ To date, in the majority of U.S. Food and Drug Administration (FDA)-approved systemic rAAV gene therapy clinical trials, the enrollment criteria have been set at either <1:10 αAAV neutralizing Ab (NAb) titer or <1:100 total αAAV-IgG titer, which would exclude a significant number of patients for the initial dosing and all individuals who need re-administration.

Our previous studies in non-human primates (NHPs) showed that low levels of pre-existing αAAV9-IgG (<1:50) had no detectable impact on tissue transgene expression following systemic rAAV9 vector delivery.^12^ Notably, a higher level of pre-existing αAAV9 Abs blocked transduction in the liver, but had very limited impact on transduction in the CNS and most of the tested different tissues in NHPs after injection of an intravenous (i.v.) vector.^12^ Given the relatively broad, but not necessarily reciprocal, cross-reactivity among AAV serotypes, some pre-existing αAAV9 Abs may be attributable to infections with other AAV serotypes.^15^, ^25^ It is unclear regarding the lowest levels of pre-existing Abs that have a significant impact on rAAV after systemic delivery, particularly in the context of Abs arising from natural AAV infection.

For clinical relevance in this study, we used NHPs that have naturally occurring pre-existing αAAV Abs similar to humans to investigate the impact of αAAV9 Ab levels on the transduction efficiency of systemically delivered rAAV9 vector. Our data demonstrate that total αAAV9-IgG at ≤1:400 had no detectable impact on tissue transduction efficiency following an i.v. rAAV9 vector delivery.

## Results

To determine the impact of pre-existing αAAV Abs on systemic rAAV gene delivery, we performed i.v. injection of rAAV9 vector in 2- to 2.6-year-old *Macaca cynomolgus* monkeys that presented various levels of pre-existing αAAV9 Abs in serum screening by ELISA for total Ig against AAV9. In general, an αAAV Ab titer <1:50 was considered negative. Seven animals with 1:100–1:6,400 titers of αAAV9-IgG were selected and used in this study. All seven animals were treated with an i.v. injection of 1 × 10^13^ vg (vector genomes)/kg rAAV9/chicken β-actin/codon-optimized human α-N-acetylglucosaminidase coding sequence cDNA (rAAV9-CBA-h*NAGLU*^op^) vector. The animals were observed daily for well-being and behavior after the vector treatment. Necropsy was performed at 5 weeks after injection (p.i.) for tissue analyses of transgene expression, vector biodistribution, and histopathology. Tissues from four NHPs from a previous study were used as controls, including two non-treated animals and two animals that had been αAAV9 Ab negative at the time that they received an i.v. injection of a similar rAAV9 vector, rAAV9-cytomegalovirus (CMV)-h*NAGLU* (produced by SAB Tech, Philadelphia, PA), at 1 × 10^13^ vg/kg and had been terminated at 6 weeks or 3 months p.i.^12^ Table 1 summarizes the study design.

**Table 1:** Study Design: Systemic Delivery of rAAV9-CBA-h*NAGLU*^op^ in NHPs

NHP	Sex	Age (Years)	αAAV9-IgG	Vector (vg/kg)	Necropsy Time (p.i.)
H1	M	2.6	1:1,600	1 × 10^13^	5 weeks
H2	F	2.5	1:6,400	1 × 10^13^	5 weeks
H3	F	2.4	1:800	1 × 10^13^	5 weeks
L1	M	2.1	1:400	1 × 10^13^	5 weeks
L2	F	2.4	1:200	1 × 10^13^	5 weeks
L3	M	2.4	1:100	1 × 10^13^	5 weeks
L4	F	2.4	1:200	1 × 10^13^	5 weeks
VC1	F	10.5	1:32	1 × 10^13^	6 weeks
VC2	M	11.0	–	1 × 10^13^	3 months
NT1	F	10.3	1:100	saline	n/a
NT2	M	2.3	1:50	saline	n/a

H1 to -3: NHPs with high levels of pre-existing αAAV9-IgG (1:800–1:6,400); L1 to -4: NHPs with low levels of pre-existing αAAV9-IgG (1:100–1:400); VC1 and -2: control tissues from two NHPs treated with a similar rAAV9-CMV-h*NAGLU* vector in a previous study; NT1 and -2: control tissues from two non-treated NHPs used in a previous study.

### Differential Impacts of Pre-existing αAAV9 Abs on the Transduction Efficiency of a Systemically Delivered rAAV9 Vector

**Figure 1:**
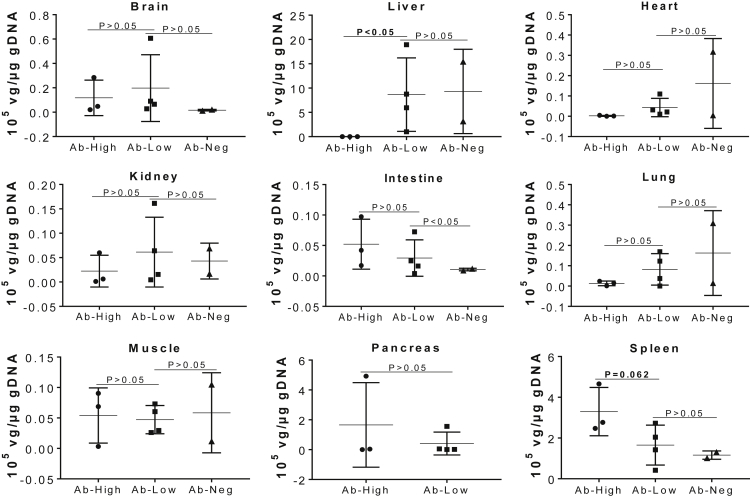
Impact of Pre-existing αAAV9 Abs on the Biodistribution of Vector Genome in NHPs after an i.v. Injection of rAAV9 Vector Seven NHPs (age, 2–2.6 years) with different levels of pre-existing αAAV9 Abs were treated with an i.v. injection of 1 × 10^13^ vg/kg rAAV9-CBA-h*NAGLU*^op^ vector. Necropsies were performed for tissue analyses at 1 month p.i. Tissues were assayed by qPCR for level of vector genome (vg). Data are expressed as 10^5^ vg/μg gDNA. Ab-High: tissues from animals with 1:800–1:6,400 serum total αAAV9-IgG (n = 3); Ab-Low, tissues from animals with 1:100–1:400 serum total αAAV9-IgG (n = 4); Ab-Neg, tissues from αAAV9-IgG-negative NHPs from a previous study, treated with rAAV9-h*NAGLU* vector (n = 2).

Real-time qPCR was performed to determine the amount of rAAV9-CBA-h*NAGLU*^op^ vector entering the CNS versus the somatic tissues. Figure 1 shows the differential biodistribution of vgs in different tissues following an i.v. vector injection in NHP subjects, with or without pre-existing αAAV9 Abs. Notably, no detectable differences were observed in vg levels in the brain or any tested peripheral tissues in animals with ≤1:400 titers of pre-existing αAAV9-IgG (Figure 1). In general, in these animals, the highest vector concentration was detected in the liver, followed by the spleen, heart, brain, and other tissues (Figure 1).

In three NHPs that had relatively higher serum αAAV9-IgG titers (1:800–1:6,400), transduction in the liver, as measured by vg copy numbers, was essentially ablated. (Figure 1). However, no significant differences were detected in most of the tested somatic tissues, including brain, heart, lung, kidney, intestine, muscle, and pancreas, among all seven NHP subjects with different titers of pre-existing αAAV9-IgG (Figure 1). These data suggest that the high pre-existing αAAV9 Abs have less impact on AAV9 transduction in the CNS and most of the somatic tissue than in the liver.

Notably, unlike other tissues, the vg levels in the spleen in the three animals with 1:800–1:6,400 pre-existing αAAV9-IgG trended higher (p = 0.062) than in the animals with 1:400 or lower titers (Figure 1), similar to our previously published data.^12^

**Figure 2:**
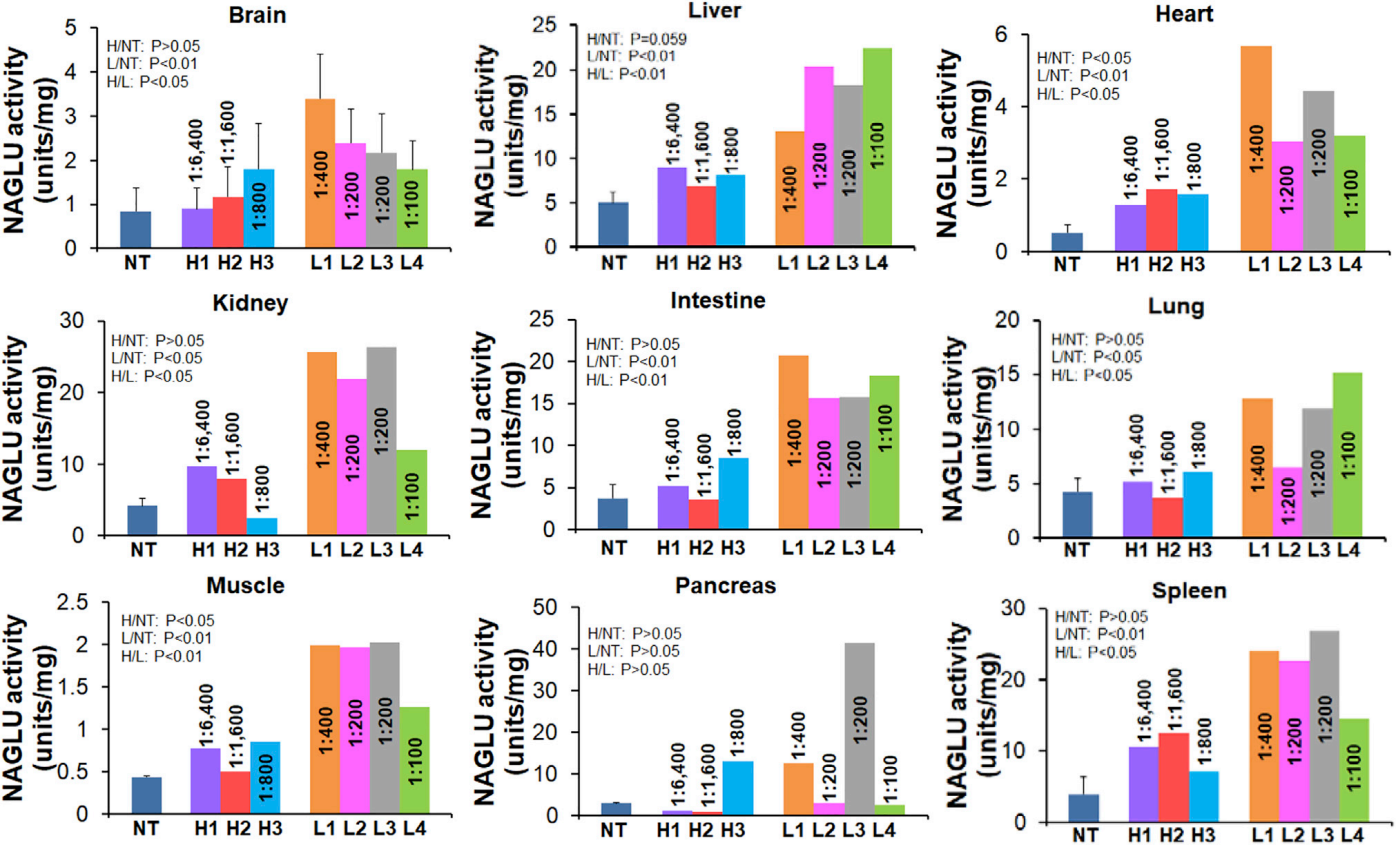
Impact of Pre-existing αAAV9 Abs on Transgene Expression Levels in NHPs after an i.v. Injection of rAAV9 Vector Seven NHPs (age, 2–2.6 years) with different levels of pre-existing αAAV9 Abs were treated with an i.v. injection of 1 × 10^13^ vg/kg rAAV9-CBA-h*NAGLU*^op^ vector. Necropsies were performed for tissue analyses at 1 month p.i. Tissues were assayed for NAGLU activity, expressed as U/mg protein: 1 U = 1 nmol 4MU released/h. H1–3, tissues from animals with 1:800–1:6,400 serum total αAAV9-IgG (n = 3); L1–4, tissues from animals with 1:100–1:400 serum total αAAV9-IgG (n = 4); NT, tissues from non-treated control animals from a previous study (n = 2).

**Figure 3:**
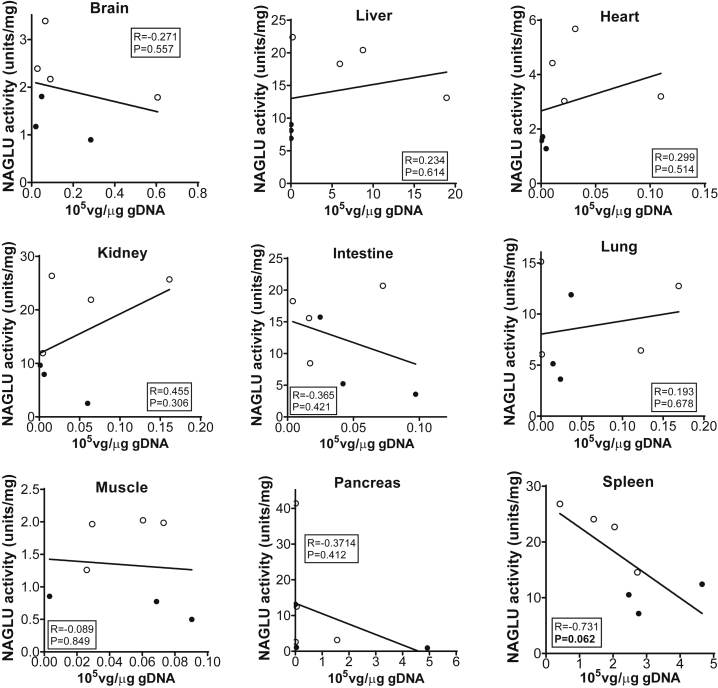
Correlation of Tissue Vector Genome and NAGLU Activity in NHPs with Different Levels of Pre-existing αAAV9 Abs after an i.v. Injection of rAAV9 vector Seven NHPs (age, 2–2.6 years) with different levels of pre-existing αAAV9 Abs were treated with an i.v. injection of 1 × 10^13^ vg/kg rAAV9-CBA-h*NAGLU*^op^ vector. Necropsies were performed for tissue analyses at 1 month p.i. Tissues were assayed for vector genome (10^5^ vg/μg gDNA) and NAGLU activity (U/mg protein). Data were analyzed for correlation between tissue vg and NAGLU activity. Solid circles, tissues from animals with 1:800–1:6,400 serum total αAAV9-IgG (n = 3); empty circles, tissues from animals with 1:100–1:400 serum total αAAV9-IgG (n = 4).

Using the NAGLU enzyme activity assay, we observed significantly lower NAGLU activity levels in all tested tissues in the three NHPs with 1:800–1:6,400 pre-existing αAAV9-IgG, in comparison to those in animals with 1:400 or lower (Figure 2), although the tissue NAGLU activity levels in animals with 1:800–1:6,400 αAAV9-IgG remained above normal wild-type (WT) levels (Figure 2). In general, the tissue NAGLU activity levels correlated with the vector biodistribution data, with the exception of the spleen (Figures 1 and 2), though the differences in tissue vg levels were not significant in the majority of the tested tissues (Figure 1). Although tissue vg copy number and NAGLU activity level appeared to correlate in most of the tissues, further correlation analysis did not reach statistical significance, though the negative correlation in the spleen approached significance. Similar to our previously published data, we saw higher vector copy numbers (p = 0.062; Figure 1), but lower NAGLU activity, in spleens from the animals with ≥1:800 αAAV9 Abs, compared with the animals with ≤1:400 αAAV9 Abs (p < 0.05; Figures 2 and 3). This suggests that pre-existing αAAV9 immunity may have driven higher levels of phagocytosis by splenocytes.

**Figure 4:**
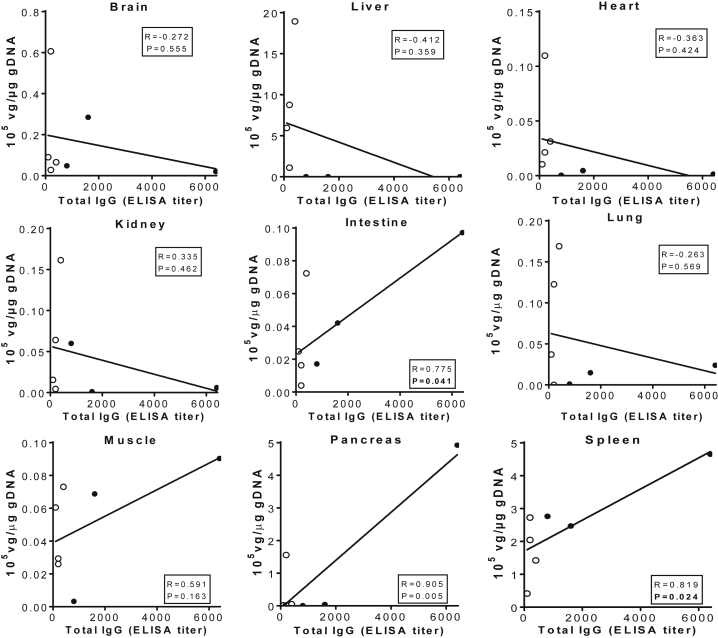
Correlation between Vector Genome Biodistribution and Serum Levels of Pre-existing αAAV9 Abs in NHPs Treated with an i.v. Injection of rAAV9 Vector Seven NHPs (age 2–2.6 years) with different levels of pre-existing αAAV9 Abs were treated with an i.v. injection of 1 × 10^13^ vg/kg rAAV9-CBA-h*NAGLU*^op^ vector. Necropsies were performed for tissue analyses at 1 month p.i. Data were analyzed for correlation between tissue vg (10^5^ vg/μg gDNA) and serum levels of pre-existing αAAV9-Abs (ELISA titer). Solid circles, tissues from animals with 1:800–1:6,400 serum total αAAV9-IgG (n = 3); empty circles, tissues from animals with 1:100–1:400 serum total αAAV9-IgG (n = 4).

Further correlation analyses showed a trend toward negative correlation between serum pre-existing αAAV9 Abs and tissue AAV vg copy levels in the brain, liver, heart, kidney, and lung (Figure 4), though it did not reach statistical significance (p > 0.05). These data further support a limited loss in transduction efficiency in most of these tissues in the presence of higher αAAV9 Abs (Figure 1). The lack of statistical significance of the negative correlation between αAAV9 Ab titers and liver vg copy numbers appeared to be due to high individual variation in the low-αAAV9-Abs group animals (Figure 4), as was also observed in Ab^−^ controls (Figure 1). Interestingly, we observed a significant positive correlation between pre-existing αAAV9 Abs and vg copy number in the spleen (p = 0.024), as well as the intestine (p = 0.041) and pancreas (p = 0.005), and non-significant correlation in muscle (p = 0.163; Figure 4).

**Figure 5:**
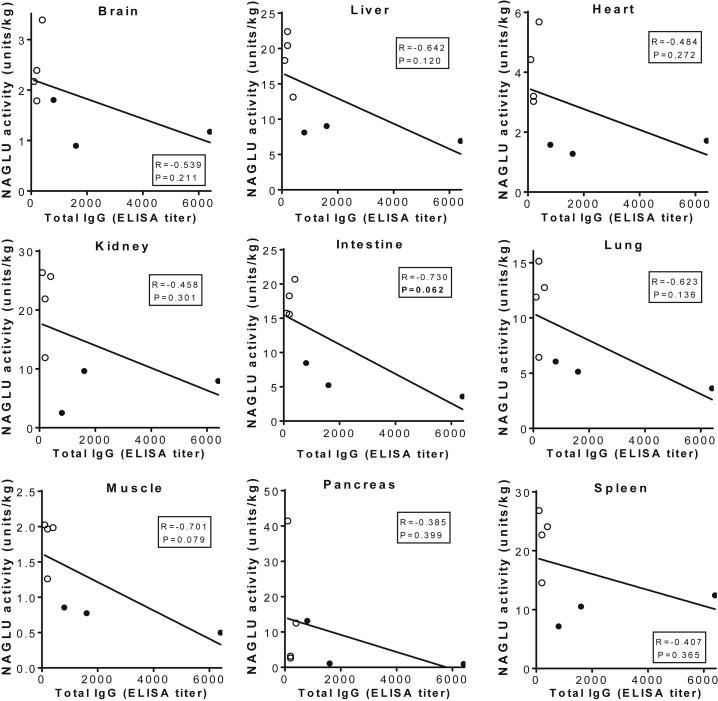
Correlation between Tissue NAGLU Activity and Serum Levels of Pre-existing αAAV9 Abs in NHPs Treated with an i.v. Injection of rAAV9 Vector Seven NHPs (age, 2–2.6 years) with different levels of pre-existing αAAV9 Abs were treated with an i.v. injection of 1 × 10^13^ vg/kg rAAV9-CBA-h*NAGLU*^op^ vector. Necropsies were performed for tissue analyses at 1 month p.i. Data were analyzed for correlation between tissue NAGLU activity (U/mg) and serum levels of pre-existing αAAV9 Abs (ELISA titer). Solid circles, tissues from animals with 1:800–1:6,400 serum total αAAV9-IgG (n = 3); empty circles, tissues from animals with 1:100–1:400 serum total αAAV9-IgG (n = 4).

Furthermore, our data showed negative correlation of serum pre-existing αAAV9 Abs to tissue NAGLU activity in all tested tissues, though it did not reach statistical significance (Figure 5). Notably, near-significant negative correlations were observed between serum αAAV9 Abs and NAGLU activity in the intestine (p = 0.062) and muscle (p = 0.079; Figure 5).

### No Observable Adverse Events in NHPs after an i.v. Injection of rAAV9-CBA-h*NAGLU*^*op*^

Daily observation for the entire 5 weeks of the experiments did not reveal any adverse events or abnormalities in the rAAV9-treated subjects, suggesting that systemic administration of the rAAV9-CBA-h*NAGLU*^*op*^ vector in the presence of various titers of pre-exiting αAAV Ab at a dose of 1 × 10^13^ vg/kg posed little risk of adverse reactions for general health or neural function.

### No Detectable Systemic Toxicity in NHPs after an i.v. Infusion of rAAV9-CBA-h*NAGLU*^op^

Histopathologic examination demonstrated no treatment-related lesions in organs of major systems—cardiovascular, digestive (including liver), lymphoid, nervous (CNS [brain and spinal cord], peripheral nervous system [PNS], dorsal root ganglions [DRGs], and nerves), pulmonary, reproductive, and urinary—and selected organs (skeletal muscle and thyroid gland), indicating that the vector treatment induced no systemic toxicity. These data support the safety of i.v. injection of the rAAV9-CB-h*NAGLU*^op^ vector as a potential strategy for gene therapy in human patients.

## Discussion

The most important outcome of this study is the demonstration of the threshold at which αAAV Abs significantly diminished the transduction efficiency of systemically delivered AAV vectors in NHPs. As effective AAV gene therapy products advance to clinical application, the high prevalence of pre-existing humoral immunity against AAV in humans impedes the translation of systemic AAV gene delivery for the treatment of human diseases. Although the serum pre-existing αAAV Ab titer varies greatly among individuals,^15^, ^16^, ^24^ the enrollment criteria have been set at either <1:10, for the αAAV-NAb titer, or <1:100, for the total αAAV-IgG titer, in the majority of FDA-approved systemic AAV gene therapy clinical trials. These would exclude a significant number of patients for the initial dosing and all individuals who need re-administration. Using αAAV9-IgG as target, this study showed that low levels (≤1:400) of pre-existing AAV9 Ab did not greatly affect tissue transduction in NHPs, suggesting that systemic rAAV9-h*NAGLU*^op^ gene delivery may also be efficacious in treating patients with similarly low levels of pre-existing αAAV9 Abs. This is consistent with our previous observation in i.v. rAAV9-h*NAGLU* gene delivery in NHPs^12^ and in a hemophilia gene therapy clinical trial in one patient who had low level (1:37) of Ab to the AAV8 vector.^1^ These results support expansion of the enrollment criteria to ≤1:400 total αAAV-IgG titer for systemic rAAV gene-delivery clinical trials, to broaden patient eligibility for AAV gene therapy treatments, in this case, making more MPS IIIB patients eligible for systemic rAAV9-h*NAGLU*^op^ gene delivery. Our recent studies showed that serum total αAAV9-IgG titers are ≤1:400 in 74% of αAAV9 Abs-positive children, including MPS III patients and healthy children,^25^ supporting the potential eligibility of the vast majority of the pediatric patient population for systemic rAAV9 gene-delivery treatments. Notably, this is a critical issue because no treatments are currently available for most neurogenetic diseases, including MPS III. Further, this could also benefit patients who may need re-administration down the road after the initial AAV vector treatment, when the rAAV gene-delivery-induced αAAV Abs titer declines to 1:400 or lower.

We also demonstrated that pre-existing αAAV9 Abs at a titer >1:400 (1:800–1:6,400) blocked transduction in the liver, but had relatively limited impact on transduction in the CNS and most of the tested peripheral tissues in NHPs after an i.v. rAAV9 vector injection. This is consistent with our previous observation in a single NHP.^12^ Although the mechanisms are unclear regarding the disparity of αAAV9 Ab effects on transduction between the liver and other tissues, it may be related to the kinetics of rAAV9 CNS entry. It is possible that the initial vector-receptor binding is a rapid process in most tissues, allowing adsorption of vector before Ab-vector interaction. The liver has a double blood supply, with approximately 75% of total liver blood flow coming from the portal vein and the remainder coming from the hepatic artery. Therefore, in individuals with no or low levels of pre-existing αAAV Abs, AAV vectors enter the CNS and other peripheral organs exclusively via the arteries after an i.v. injection, whereas most of the AAV vector enters the liver through the portal vein. Because there is a natural delay in blood flow to the liver through the portal vein, compared with that from the hepatic artery, it is possible that Ab-vector binding occurs during this delay, in the presence of αAAV Abs at levels above the threshold (>1:400), leading to the clearance of the vectors in the blood flow before reaching the liver through the portal vein. Alternatively, the critical ligand for receptor binding in other tissues may be different from that for entry in the liver, such that it may be not be blocked efficiently by the Ab.

Whatever the mechanism, the possibility that the rAAV vector entering the CNS and other peripheral tissues is less susceptible to pre-existing αAAV Abs would potentially expand the patient population that could benefit from systemic AAV vector treatment in diseases where the liver is not the major target organ. This is also likely to offer significant benefit in the clinical setting, considering that almost half of the general human population is AAV9-Ab-positive at some level. Previous studies demonstrated broad Ab cross-reactivity across different AAV serotypes in humans.^15^, ^25^ It is therefore possible that the low levels of pre-existing αAAV9 Abs detected in our NHP screen are largely attributable to cross-reacting Abs from infections with other AAV serotypes, with relatively lower affinity for AAV9 capsid. For patients with high levels of pre-existing αAAV9 Abs, immunomodulation regimens will be required prior to AAV vector administration, as shown in previous studies in animal models^18^, ^26^, ^27^ and in patients with Pompe disease.^28^, ^29^

Again, an exception observed in this study was that the NHPs with high pre-existing αAAV9-IgG (>1:400) had much higher vg copies (p = 0.062) but significantly lower NAGLU activity in the spleen, compared with animals with lower αAAV9 Abs (≤1:400), indicating that the high vg in the spleen did not concordantly result in high levels of rNAGLU expression. This is consistent with our previous observation, though with only 1 NHP available.^12^ It is highly likely that most of the vector in the spleens of animals with high levels of Ab is a consequence of phagocytosis promoted by Ab binding to circulating vector as part of the initial innate immune response to vector delivery, whereas the phagocytized vectors in splenocytes were not further processed to achieve transgene expression. Therefore, immunomodulation targeting innate immunity before and after rAAV vector delivery may reduce the phagocytosis and increase the transduction efficiency of the treatment. Efforts are needed to develop a fine-tuned immune modulation regimen to eliminate the effects of phagocytosis and innate immune responses following the rAAV vector delivery.

In summary, this study demonstrated in NHPs the presence of a threshold of αAAV9 Abs for diminishing the transduction efficiency of systemically delivered AAV9 vectors, supporting the expansion of patient eligibility to include patients with ≤1:400 serum total αAAV9-IgG for AAV gene therapy treatment. We also demonstrated that αAAV9 Abs above the 1:400 threshold blocked transduction in the liver but had a more limited impact on the CNS and most of the tested peripheral tissues. This study also suggested that higher pre-existing αAAV9 Abs promotes phagocytosis by splenocytes and that phagocytized AAV vectors are not processed for transgene expression, supporting the possibility that suppressing innate immunity in individuals with high AAV Abs may have positive impacts on the transduction efficiency of systemic AAV gene delivery.

## Materials and Methods

### Recombinant AAV Viral Vectors

An rAAV vector plasmid was constructed and used to produce a conventional single-strand rAAV9-CBA-h*NAGLU*^*op*^ viral vector, a therapeutic vector for treating MPS IIIB. The vg contained minimal elements required for transgene expression, including AAV2 terminal repeats, a hybrid human CMV-CBA promoter, an SV40 splice signal, h*NAGLU*^op^, and a polyadenylation signal. The rAAV9 viral vectors were produced by SAB Tech in HEK293 cells by transient transfection and purified by double cesium chloride gradient ultracentrifugation. The purified viral vectors were titrated by using PAGE and silver staining and confirmed by dot blot hybridization.

### Animals and i.v. Vector Delivery

Animal procedures were approved by the Institutional Animal Care and Use Committee at the Research Institute at Nationwide Children’s Hospital (NCH-RI). All animals were housed and cared for at Nationwide Children’s Hospital, in accordance with the *Guide for the Care and Use of Laboratory Animals* (DHHS Publication No. [NIH] 85-23]. Seven *Macaca cynomolgus* monkeys, 2–2.6 years of age, were obtained from Worldwide Primates (Miami, FL) (Table 1). Prior to the experiments, the animals’ sera were screened with an ELISA for pre-existing antibodies to the AAV9 capsid.

For vector delivery, veterinary staff anesthetized the subjects with an intramuscular (i.m.) (6 mg/kg). The subjects were then treated by i.v. injection of 1 × 10^13^ vg/kg rAAV9-CBA-h*NAGLU*^*op*^ vector (in 5 mL saline) via the cephalic vein. Upon recovery, the subjects were returned to their housing and observed daily for well-being and behavior throughout the experiments.

### Blood and Tissue Analyses

Blood draws were performed prior to vector injection and before necropsy at 5 weeks p.i. The subjects were euthanized by veterinary staff at 5 weeks p.i. for tissue analyses by i.v. injection of Euthasol (1 mL/10 lb). Brain, spinal cord, and multiple somatic tissues (liver, kidney, spleen, heart, lung, intestine, stomach, pancreas, and skeletal muscle) were harvested, either on dry ice and stored at −80°C, or in 4% paraformaldehyde at 4°C. As previously described,^12^ each brain was divided into two hemispheres along the midline and then into multiple coronal slabs. Each slab from one sphere was further divided into matrices with 12–14 sections, and each section was harvested on dry ice and stored at −80°C. Brain slabs from the other sphere were fixed and stored in 4% paraformaldehyde.

#### *NAGLU* Activity Assay

Tissue samples were assayed for NAGLU enzyme activity according to a published procedure, with minor modifications.^30^, ^31^ The assay measures 4-methylumbelliferone (4MU), a fluorescent product formed by hydrolysis of the substrate 4-methylumbellireyl-N-acetyl-α-d-glucosaminide. Tissue NAGLU activity is expressed as U/mg protein. Serum or CSF NAGLU activity is expressed as U/mL: 1 U = 1 nmol 4MU released per hour at 37°C.

#### Binding ELISA

Serum samples were assayed by a binding ELISA to determine the levels of total IgG against AAV9, according to previously published procedures.^12^ Empty capsid particles of AAV9 were obtained from SAB Tech and used as an antigen for the assay.

Briefly, 96-well plates were coated with 1 × 10^10^ particles/mL of empty AAV9 capsids in carbonate coating buffer (antigen-positive [ag^+^]) and carbonate coating buffer only (antigen-negative [ag^−^]) for each sample. Following incubation overnight at 4°C, the plates were washed with PBS containing 0.1% Tween-20 (pH 7.4; PBS-T) and blocked for 1 h with blocking buffer, PBS-T, and 5% dry milk. Two-fold serial dilutions (beginning at 1:50) of serum samples in blocking buffer were added to the plates and incubated at room temperature for 1 h. The plates were washed with PBS-T and then incubated with horseradish-peroxidase-conjugated anti-human IgG (Sigma-Aldrich, St Louis, MO) for 1 h at room temperature. After being washed with PBS-T, the plates were developed with 3,3′,5,5′-tetramethylbenzidine (TMB; Sigma-Aldrich, St Louis, MO) at room temperature for 5 min. The reaction was stopped by adding 1 M sulfuric acid. The absorbance was read at 450 nm on a plate reader. Serum total αAAV-IgG levels were expressed as an ELISA titer, based on the following calculation: (OD450-ag^+^ − OD450-ag^−^)/OD450-ag^−^. Values ≥2 were considered Ab positive.

#### Real-Time qPCR

Total DNA was isolated from tissue samples of treated and non-treated NHP subjects using QIAGEN DNeasy columns. Brain DNA was isolated from cerebral cortex, hippocampus, and hypothalamus. The DNA samples were analyzed by qPCR, using Absolute Blue QPCR Mix (Thermo Scientific) and Applied Biosystems 7000 Real-Time PCR System, following the procedures recommended by the manufacturer. Taqman primers specific for the CMV/CBA promoter were used to detect rAAV vgs: forward: 5′-GGCAGTACATCAAGTG TATC-3′; reverse: 5′-ACCAATGGTAATAGCGATGAC-3′; probe: [6∼FAM]AATGACGGTAAATG GCCCGC[BHQ1a∼6∼FAM]. Genomic DNA was quantified in parallel samples using β-actin-specific primers: forward: 5′-CCTTCCGTCCTCAGATCATT-3′; reverse: 5′-CTTGCTGATCCACAT CTGCT-3′; probe: [6∼FAM]CATCCTGGCCTCGCTGCCA[BHQ1a∼6∼FAM]. Genomic DNA from non-treated NHP tissues was used as controls for background levels and absence of contamination.

#### Histopathology

All collected tissues were processed to produce paraffin-embedded sections (4 μm) and then stained with H&E by the Morphology Core at NCH-RI. The sections were examined for histopathology under a microscope.

### Statistical Analyses

Means, SD, and unpaired Student’s t test were used to analyze serum Ab levels, tissue NAGLU activity, and vg bio-distribution data. Correlation analyses were performed to assess the associations between the different variables. The significance level was set at p ≤ 0.05. Prism 8 was used for data analyses.

## Author Contributions

A.S.M. and R.J.P. performed the tissue analyses and serum Ab assays. L.G. performed AAV vector injections. T.A.B. performed data analyses and prepared the manuscript. H.F. designed and led the study.

## Conflicts of Interest

The authors declare no competing interests.
